# Addressing Patients’ Alcohol Consumption–A Population-Based Survey of Patient Experiences

**DOI:** 10.3389/ijph.2021.1604298

**Published:** 2021-11-02

**Authors:** Torgeir Gilje Lid, Nadine Karlsson, Kristin Thomas, Janna Skagerström, Amy O'Donnell, Latifa Abidi, Per Nilsen

**Affiliations:** ^1^ Stavanger University Hospital, Stavanger, Norway; ^2^ Faculty of Health Sciences, University of Stavanger, Stavanger, Norway; ^3^ Department of Health, Medicine and Caring Sciences, Linköping University, Linköping, Sweden; ^4^ Region Ostergotland, Linköping, Sweden; ^5^ Faculty of Medical Sciences, Newcastle University, Newcastle Upon Tyne, United Kingdom; ^6^ Faculty of Health, Medicine and Life Sciences, Maastricht University, Maastricht, Netherlands

**Keywords:** prevention, healthcare, alcohol, brief intervention, implementation, population survey

## Abstract

**Objectives:** To identify the proportion of the population that had experienced that alcohol was addressed in health care the previous year, to explore experiences and perceived effects of addressing alcohol, and to investigate the proportion of risky drinkers in the population.

**Methods:** Cross-sectional national web-based survey with 1,208 participants. Socio-demographic data, alcohol consumption (AUDIT-C), and experiences with alcohol conversations were investigated.

**Results:** Approximately four in five respondents had visited health care the past 12 months, and one in six reported having experienced addressing alcohol. Women and older respondents were less likely to report having experienced alcohol conversations compared to other groups. Risky drinkers were not more likely to have experienced an alcohol conversation, but reported longer duration of alcohol conversations and more frequently perceived addressing alcohol as awkward or judgmental. Almost a third of respondents were classified as risky drinkers.

**Conclusion:** The proportion experiencing addressing alcohol in routine health care is low, also among risky drinkers, and risky drinkers more frequently experienced the conversations as judgmental. More sensitive and relevant ways of addressing alcohol in health care is needed.

## Introduction

Alcohol constitutes a major public health problem worldwide, and addressing alcohol use in routine health care (i.e., primary or secondary health care other than alcohol or drug treatment settings) remains a considerable challenge [1–4]. Whilst the literature shows small-to-moderate effect sizes on average for screening and brief intervention (SBI) in routine health care, the potential public health impact is high if widely implemented [5–7]. Addressing alcohol typically involve screening for risky or harmful drinking, information on health risks of alcohol and advice to help patients cut down (brief interventions) [7]. The aim is to reduce alcohol consumption and related harm in patients not actively seeking help for their drinking, and occurs when the main purpose of the health care visit is something else [7].

The implementation of alcohol SBI in everyday practice has been low, due to barriers related to the views of individual health care professionals (HCPs), as well as a range of organisational factors [2, 8–10]. One important barrier concerns HCPs’ preconceptions of alcohol as a sensitive and stigmatising topic, potentially affecting the doctor-patient relationship if brought up [8, 11, 12]. Few studies have investigated patient’s experiences of addressing alcohol in routine health care, although recent studies in Sweden, England and the Netherlands suggest that patients are more open to addressing alcohol than HCPs expect [13–15]. An interview study 1 week after a hospital stay, of patients without previous alcohol use disorder but admitted with an alcohol-related condition, found that a majority of participants were positive to HCPs raising the topic of alcohol and offering a brief intervention by an alcohol care team [16]. There is also evidence that elderly patients, patients with lower socioeconomic status (SES), and risky drinkers, may be less positive to HCPs raising the topic of alcohol [13, 16].

Until 1990, alcohol consumption in Norway was low compared to other parts of Europe, with 4–5 L pure alcohol sold per person aged 15 years and older. By 2018, the annual per capita pure alcohol litres sold had increased to over 6 L per person, excluding non-registered consumption [17]. This increase may partly be related to increased affordability (higher incomes and lower alcohol taxation) and increased availability (three-fold increase in wine monopoly outlets from late nineties to 2017) [18]. Alcohol guidelines in Norway have focused on specific patient groups, e.g., pregnant women, patients with drug problems and comorbid mental disorders, and patients in medical and surgical hospital wards [19, 20]. Further, the Norwegian Ministry of Health and Care Services has issued mandates ordering hospitals to implement routine screening for alcohol and drug problems in medical and surgical wards, and, when necessary, refer patients to specialised treatment [21]. These mandates, first issued in 2013, did not include any specific guidance on how to address alcohol, and the uptake of routine screening for alcohol and drug problems in hospitals is still low (unpublished data, article submitted).

There is limited knowledge of how alcohol is addressed in routine health care and of the effects on drinking patterns, quality of life and health status. However, experiencing an alcohol conversation may positively affect risky drinker’s attitudes towards such preventative conversations [22]. We need a better understanding of patient’s perspectives and experiences, but nation-wide data on the proportion of risky drinkers in Norway is lacking, and no study has explored the proportion of the population who has experienced addressing alcohol in health care, how it was experienced, and the perceived effects of addressing alcohol.

The aims of this study were: 1) to identify the proportion of the Norwegian population that had an alcohol in health care during the previous year; 2) to explore individual’s experiences with these conversations; and 3) to investigate the proportion of risky drinkers in the population.

## Methods

### Study Population

A cross-sectional, nationally representative sample of 6,000 adults aged 18–88 years was randomly drawn from a web-panel of 30,000 participants (i.e., representative of age, sex, region of residence and size of municipality). Of these, 1,208 responded before recruitment was terminated. Response Analyse, a company specializing in survey research, performed the survey and the comparisons with national data on the time of the survey (December 2018). The gender balance of the responders was identical to the national population (49.7% women). There were fewer younger respondents than expected (18–24 years, 11.5% in the survey vs. 14.7% nationally) and slightly more responders in the oldest age group (65+ years, 20.8% in the survey vs. 19.4% nationally). For other age groups, the differences were less than 1%.

### Design and Data Collection

This study is part of an international project exploring attitudes towards and experiences with alcohol conversations in routine health care in Europe [13–15]. We aimed to recruit a minimum of 1000 respondents, based on population size (5.3 million inhabitants in 2018), and margins of error. Closing the survey was a manual procedure, and 1208 individuals had responded when the survey was closed after 2 weeks. Data was collected from the 7th to the December 20th, 2018 by means of a web-based questionnaire, accessed by the participants via a unique link provided in an e-mail invitation. No reminders were sent. Participants had previously consented to be part of the web panel of 30.000, and they consented to this specific study by actively accessing the link and answering the questionnaire.

### Questionnaire

We translated the questionnaire from English, and repeatedly tested and adjusted by comparing with the Swedish version. The questionnaire consisted of 16 questions regarding socio-demographic data, alcohol consumption, attitudes towards and experiences with alcohol conversations in routine health care. Education was reported in one of five categories (basic or secondary school; 1–3 years college/university; 4–5 years college/university; six or more years of college/university; PhD or specialist qualification from college/university). We collapsed the three highest categories into one (four or more years of college/university) in the analyses. Current occupational status was reported in one of eight categories (employed, student, unemployed, long-term sick leave, retired, parental leave or other leave, self-employed, other). Sociodemographic characteristics, health care visits and drinking status are reported in Table 1.

**TABLE 1:** Respondent’s socio-demographic characteristics, health care visits and conversations about alcohol in health care, and drinking characteristics (weekly alcohol consumption g/week; Frequency of HED occasions) in implementation of Acohol Prevention Study, Norway 2018.

Variables	Total (n = 1208) n (%)	Male (n = 551) n (%)	Female (n = 657) n (%)
Age (*n* = 1208)			
Mean age (SD)	48.8 (15.9)	50.8 (16.2)	47.1 (15.5)
Age categorical (*n* = 1208)			
<29 years	165 (13.7)	62 (11.3)	103 (15.7)
30–39 years	208 (17.2)	81 (14.7)	127 (19.3)
40–49 years	240 (19.9)	110 (20.0)	130 (19.8)
50–59 years	237 (19.6)	109 (19.8)	128 (19.5)
60–60- years	246 (20.4)	117 (21.2)	129 (19.6)
≥70- years	112 (9.3)	72 (13.1)	40 (6.1)
Education (*n* = 1191)			
Basic or secondary school	356 (29.9)	165 (30.6)	191 (29.3)
College or university (1.-3 years)	366 (30.7)	175 (32.4)	191 (29.3)
College or university (4 years or more)	469 (39.4)	200 (37.0)	269 (41.3)
Occupation (*n* = 1207)			
Employed	804 (66.6)	384 (69.8%)	420 (63.9%)
Student	62 (5.1)	25 (4.5%)	37 (5.6%)
Unemployed	17 (1.4)	5 (0.9%)	12 (1.8%)
Sick-listed	22 (1.8)	6 (1.1.%)	16 (2.4%)
Retired	237 (19.6)	116 (21.1%)	121 (18.4%)
Parental leave	14 (2.1%)	0 (0.0%)	14 (2.1%)
Other	51 (4.2%)	14 (2.5%)	37 (5.6%)
Marital status (*n* = 1208)			
Married/living together	780 (64.6%)	388 (70.4%)	392 (59.7%)
Relationship but living apart	81 (6.7%)	36 (6.5%)	45 (6.8%)
Single	347 (28.7%)	127 (23.0%)	220 (33.5%)
Health care visits in the last 12 months (*n* = 1208)			
2 or more visits	608 (50.3%)	236 (42.8%)	372 (56.6%)
1 visit	351 (29.1%)	165 (29.9%)	186 (28.3%)
No visit	249 (20.6%)	150 (27.2%)	99 (15.1%)
Conversations about alcohol in health care in the last 12 months (*n* = 959)			
2 or more conversations	43 (4.5%)	22 (5.5%)	21 (3.8%)
1 conversation	118 (12.3%)	51 (12.7%)	67 (12.0%)
No conversation	798 (83.2%)	328 (81.8%)	470 (84.2%)
Alcohol, consumption (NY see SAP)			
Weekly alcohol consumption (number of glass per week; mean+/-SD) (n = 1208)	3.5 (5.2)	4.7 (6.3)	2.6 (3.7)
HED (q8; n = 1088)			
Never	185 (17.0%)	70 (13.9%)	115 (19.7%)
Less than once a month	541 (49.7%)	242 (47.9%)	299 (51.3%)
Once a month	167 (15.3%)	77 (15.2%)	90 (15.4%)
2–3 times a month	116 (10.7%)	63 (12.5%)	53 (9.1%)
1–2 times a week	62 (5.7%)	41 (8.1%)	21 (3.6%)
3–4 times a week	15 (1.4%)	10 (2.0%)	5 (0.9%)
7 times a week	2 (0.2%)	2 (0.4%)	0 (0.0%)
HED (q8 dichotomized acc to HED occasions; *n* = 1088)			
Low risk	726 (66.7%)	312 (61.8%)	414 (71.0%)
Risky drinkers	362 (33.3%)	193 (38.2%)	169 (29.0%)
Drinking characteristics in 3 levels (*n* = 1208)			
Abstainers	120 (9.9%)	46 (8.3%)	74 (11.3%)
Low risk drinkers	719 (59.5%)	310 (56.3%)	409 (62.3%)
Risky drinkers	369 (30.5%)	195 (35.4%)	174 (26.5%)

Alcohol consumption was measured using the three-item Alcohol Use Disorders Identification Test–Consumption (AUDIT-C), and three alcohol consumption categories were constructed: abstainers; low risk drinkers; and risky drinkers [23]. AUDIT-C consists of the first three questions in AUDIT, measuring alcohol consumption. The complete Alcohol Use Disorders Identification Test (AUDIT) consists of ten questions, assessing harm and addiction in addition to consumption, but with ten questions, it was less likely to be embedded into general history questionnaires [23]. Respondents reporting no alcohol consumption in the previous 12 months were classified as abstainers. Low risk drinking was defined as a weekly consumption of up to 14 standard drinks per week for men and up to nine drinks per week for women, and/or heavy episodic drinking (HED), on no more than a monthly basis. HED was defined as consuming five drinks or more per occasion for men or four drinks or more for women. Risky drinking was defined as consumption above these levels. One standard drink equated to 12 g of pure alcohol, as in previous research [15].

One item asked participants weather they had visited their health care centre in the past 12 months, with response options: “no”, “yes, once” or “yes, more than once”. Respondents who answered “no” did not receive further questions. The question concerning whether alcohol use was addressed in health care in the past 12 months was answered with “no”, “yes, once” or “yes, more than once”. Respondents who answered “no” did not receive further questions.

One item investigated the duration of alcohol conversations including four response options: less than 1 min, 1–4 min, 5–10 min, and more than 10 min. In Table 2, the categories 5–10 min and more than 10 min has been collapsed. Respondents with more than one conversation about alcohol were asked to report the average duration. To investigate perceived contents of the conversation, the respondents were asked to respond “yes” or “no” to each of five different items: information about health consequences; how much alcohol they usually drank; whether they wanted to cut down; advice on how to cut down; and written information about alcohol. Perceptions of the alcohol conversations were assessed by four items with a four-item Likert response scale, ranging from “do not agree” to “agree completely”. The four statements included: “it provided valuable knowledge”, “it was informative”, “it was routine”, “it felt awkward”, “it was irritating”, “it was offensive”, and “it felt judgmental”.

**TABLE 2:** Health care visits and conversations about alcohol in the healthcare in Norway among the abstainers, low risk drinkers, and risky drinkers. Implementation of Acohol Prevention Study, Norway 2018.

Variables	Total n (%)	Abstainers n (%)	Low risk drinkers n (%)	Risky drinkers n (%)	p-value (chi-squared test)
Health care visits in the past 12 months (*n* = 1208)					
2 or more visits	608 (50.3%)	63 (52.5%)	359 (49.9%)	186 (50.4%)	0.958
1 visit	351 (29.1%)	34 (28.3%)	207 (28.8%)	110 (29.8%)	
No visit	249 (20.6%)	23 (19.2%)	153 (21.3%)	73 (19.8%)	
Conversation about alcohol in health care in the past 12 months (*n* = 959)					
2 or more conversations	43 (4.5%)	5 (5.2%)	22 (3.9%)	16 (5.4%)	0.654
1 conversation	118 (12.3%	9 (9.3%)	69 (12.2%)	40 (13.5%)	
No conversation	798 (83.2%)	83 (85.6%)	475 (83.9%)	240 (81.1%)	
Duration of conversation about alcohol (*n* = 161)					
<1 min	109 (67.7%)	10 (71.4%)	71 (78.0%)	28 (50.0%)	0.007
1–4 min	28 (17.4%)	2 (14.3%)	13 (14.3%)	13 (23.2%)	
>5 mn	24 (14.9%)	2 (14.3%)	7 (7.7%)	15 (26.8%)	
Contents of conversation about alcohol (*n* = 161) (affirmative answers)					
a. Information about alcohol’s influence on health	23 (14.3%)	3 (21.4%)	8 (8.8%)	12 (21.4%)	0.076
b. Questions about my alcohol consumption	117 (72.7%)	11 (78.6%)	69 (75.8%)	37 (66.1%)	0.381
c. Questions re. my willingness to reduce consumption	8 (5.0%)	0 (0.0%)	2 (2.2%)	6 (10.7%)	0.047
d. Advice on how to reduce my consumption	6 (3.7%)	0 (0.0%)	1 (1.1%)	5 (8.9%)	0.038
e. Written information about alcohol	8 (5.0%)	1 (7.1%)	1 (1.1%)	6 (10.7%)	0.031
Experiences of conversation about alcohol (*n* = 161) (agreed completely or to a large degree 1–2 vs. 3–4)					
a. Provided valuable knowledge	44 (27.3%)	4 (28.6%)	23 (25.3%)	17 (30.4%)	0.793
b. Informative	61 (37.9%)	6 (42.9%)	33 (36.3%)	22 (39.3%)	0.863
c. Routine	132 (82.0%)	12 (85.7%)	78 (85.7%)	42 (75.0%)	0.242
d. Awkward	11 (6.8%)	1 (7.1%)	2 (2.2%)	8 (14.3%)	0.019
e. Irritating	11 (6.8%)	1 (7.1%)	3 (3.3%)	7 (12.5%)	0.100
f. Judgmental	18 (11.2%)	1 (7.1%)	6 (6.6%)	11 (19.6%)	0.045
Effects of conversation about alcohol (*n* = 161) (agreed completely or to a large degree 1–2 vs. 3–4)					
a. Had no effect at all	84 (52.2%)	9 (64.3%)	47 (51.6%)	28 (50.0%)	0.625
b. Made me think about my drinking	21 (13.0%)	1 (7.1%)	7 (7.7%)	13 (23.2%)	0.020
c. Gave me a better understanding of alcohol’s health risks	23 (14.3%)	2 (14.3%)	11 (12.1%)	10 (17.9%)	0.624
d. Led to increase in my drinking (q15_5)	15 (9.3%)	1 (7.1%)	4 (4.4%)	10 (17.9%)	0.023
e. Led to reduction of my drinking (q15_4)	19 (11.8%)	1 (7.1%)	7 (7.7%)	11 (19.6%)	0.079
f. Made me think about a friend’s drinking	26 (16.1%)	2 (14.3%)	13 (14.3%)	11 (19.6%)	0.679

Perceived effects of alcohol conversations were assessed with six statements, using a similar four-item Likert scale. The following statements were used: “it had no effect at all”, “it made me think about my drinking”, “it gave me a better understanding of alcohol’s health risks”, “it led to a reduction in my drinking”, “it led to an increase in my drinking”, and “it made me think about a friend’s drinking”.

### Statistical Methods

Descriptive statistics are presented as frequencies or as means and standard deviations. Differences in proportions between groups were examined with the chi-squared test or Fisher’s exact test. Logistic regressions were performed to examine associations between having had an alcohol conversation in the past 12 months and individual characteristics. The odds ratios (OR) of having had an alcohol conversation in the past 12 months were first estimated in a model adjusted for age and gender (model 1 in Table 3), then adjusted for age, gender, educational level, occupational status, marital status, drinking categories and number of healthcare visits in the past 12 months (model 2 in Table 3). Data were analysed using the statistical software IBM SPSS 25. Results were statistically significant at *p* ≤ 0.05 using two-tailed tests.

**TABLE 3:** Logistic regression of having had a conversation about alcohol in healthcare in the past 12 months in Norway in function of determinants among respondents who had visited the healthcare in the past 12 months (*n* = 959). Implementation of Acohol Prevention Study, Norway 2018.

Variables	Model I (age and sex adjusted)				Model II (multivariate)			
	N	OR^a^	95%CI	*p*-value	N	OR^b^	95%CI	*p*-value
Gender								
Male	401	1			394	1		
Female	558	0.69	(0.48–0.98)	0.04	553	0.56	(0.38–0.83)	0.004
Age								
18–29 years	121	1			120	1		
30–39 years	172	0.67	(0.40–1.14)	0.138	170	0.74	(0.40–1.39)	0.354
40–49 years	177	0.43	(0.24–0.74)	0.002	176	0.44	(0.23–0.86)	0.015
50–59 years	190	0.30	(0.17–0.54)	<0.001	188	0.30	(0.15–0.58)	0.001
60–69 years	201	0.25	(0.14–0.46)	<0.001	198	0.24	(0.11–0.53)	<0.001
≥70 years	98	0.13	(0.05–0.31)	<0.001	95	0.10	(0.03–0.34)	<0.001
Education								
Basic or secondary school	270	1			270	1		
College or university (1-3y)	297	1.07	(0.69–1.65)	0.781	297	1.14	(0.72–1.81)	0.581
College or university (≥4y)	381	0.86	(0.55–1.33)	0.491	380	0.88	(0.55–1.41)	0.588
Occupation								
Employed	619	1			613	1		
Student	45	1.71	(0.81–3.62)	0.163	45	1.41	(0.64–3.13)	0.395
Unemployed	11	0.48	(0.06–3.88)	0.490	11	0.34	(0.04–2.80)	0.314
Sick-listed	21	1.21	(0.39–3.73)	0.737	20	1.13	(0.35–3.60)	0.838
Retired	206	1.36	(0.65–2.86)	0.417	202	1.31	(0.61–2.81)	0.483
Parental leave	14	8.27	(2.63–25.97)	<0.001	14	8.62	(2.56–29.04)	0.001
Other	42	2.20	(1.05–4.63)	0.038	42	1.93	(0.88–4.23)	0.101
Marital status								
Married/living together	623	1			612	1		
Relationship but living apart	62	1.47	(0.78–2.78)	0.249	62	1.50	(0.76–2.98)	0.243
Single	274	0.90	(0.60–1.36)	0.578	273	0.83	(0.54–1.28)	0.398
Drinking categories								
Abstainers	97	1			96	1		
Low risk drinkers	566	1.13	(0.60–2.09)	0.656	560	1.56	(0.76–3.20)	0.221
Risky drinkers	296	1.36	(0.70–2.62)	0.334	291	1.93	(0.91–4.11)	0.086
Number of health care visits in the past 12 months								
1 visit	351	1			346	1		
2 or more visits	608	3.15	(2.06–4.82)	<0.001	601	3.03	(1.96–4.69)	<0.001

a ORs are adjusted for age and gender.

b ORs are adjusted for age, gender, educational level, occupation, marital status, drinking categories, and health care visits in the past 12 months.

OR, odds ratio; CI, confidence interval.

## Results

A total of 1,208 individuals responded to questions on drinking status, attitudes towards and experiences with alcohol conversations in routine health care, and their perceived effects.

Table 1 presents sociodemographic and drinking characteristics of the respondents by gender. Nearly one-third of the respondents (29.7%) were 60 years or older, and one-tenth (9.3%) were 70 years or older. Almost one third of the respondents were classified as risky drinkers (30.5%), with more than one in three men (35.4%) and just above one in four women being classified as risky drinkers (26.5%).

### Characteristics of and Determinants of Alcohol Conversations in Healthcare

Eighty percent (79.4%) of the respondents had visited health care the past 12 months, and of these, one in six (16.8%) reported having had conversations about alcohol. Two or more visits in health care the past 12 months increased the likelihood of having had an alcohol conversation three-fold (OR 3.03, *p* < 0.001) in the multivariate model, compared to one visit. More women than men had visited health care in the past 12 months (84.9% of women compared to 72.8% of men), but a higher proportion of men reported a conversation about alcohol (18.2% of men, compared to 15.8% of women). In the multivariate logistic regression women were half as likely as men to have had an alcohol conversation (OR 0.56, *p* = 0.004) (see Table 3).

Results concerning health care visits and alcohol conversations by drinking categories are presented in Table 2. There were no significant differences between drinking categories with regard to health care visits, nor any significant differences between drinking categories on having had an alcohol conversation. However, risky drinkers reported considerably longer alcohol conversations than low risk drinkers and abstainers. Fifty percent of risky drinkers who had an alcohol conversation reported that it was longer than 1 min, compared to 22% of low risk drinkers and 28.6% of abstainers (*p* = 0.007). The difference was largest for the longest conversations.

Table 3 presents the results of logistic regressions of having had an alcohol conversation, adjusted for age and sex, and a multivariate model. The odds ratio of such a conversation shows a gradual decline with increasing age compared to the youngest age group, statistically significant for all age groups older than 40 years in the multivariate model. In this model, the odds ratio increased more than eight-fold (OR 8.62, *p* = 0.001) for having a conversation about alcohol for respondents on parental leave or other leave.

### Experiences and Perceived Effects of Alcohol Conversations

Table 2 shows experiences, and the perceived effects, of alcohol conversations by drinking status. Of the risky drinkers, 66.1% (n.s.) compared to low risk drinkers (75.8%) and abstainers (78.6%) reported that questions about alcohol consumption was part of the conversation, while 10.7% of risky drinkers and 2.2% of low risk drinkers (*p* = 0.047) reported questions regarding their willingness to reduce consumption. Furthermore, 8.9% of risky drinkers and 1.1% of low risk drinkers (*p* = 0.038) had received advice on how to reduce consumption, and 10.7% of risky drinkers and 1.1% of low risk drinkers (*p* = 0.031) had received written information about alcohol.

A considerably higher proportion of risky drinkers found the alcohol conversations awkward, compared to low risk drinkers and abstainers (14.3 vs. 2.2% and 7.1%, *p* = 0.019). The same pattern is evident for participants perceiving the conversations as judgmental (19.6 vs. 6.6% and 7.1%, *p* = 0.045). Almost a quarter of risky drinkers (23.4%) reported that the alcohol conversations made them think about their drinking, compared to around 7% for low risk drinkers and abstainers (*p* = 0.020). However, 17.9% of risky drinkers reported that the conversation led to an increase in their drinking, compared to 4.4% of low risk drinkers and 7.1% of abstainers (*p* = 0.023). Other experiences and perceived effects were not significant by drinking status.

Additional analyses were performed of having had an alcohol conversation and experiencing a positive effect (“it made me think about my drinking”, “it gave me a better understanding of alcohol’s health risks”, “it led to a reduction in my drinking”). A logistic regression applying the same definition of positive effect as in Table 2 (1 = “agree completely” or 2 = “agree to a large extent” vs. 3 = “agree to some extent” or 4 = “do not agree”) did not yield significant results. In a new logistic regression where positive effect was defined as either 1, 2 or 3 (“agree completely”, “agree to a large extent”, “agree to some extent”) was then performed. In the multivariate model, women had a lower odds ratio of having had a conversation in healthcare and having reported a positive effect, compared to men (OR 0.26, *p* = 0.002). Risky drinkers were more likely to report a positive effect of their alcohol conversation compared to abstainers (OR 5.49, *p* = 0.035). These results are not presented in a table because of the wider definition of positive effect compared to Table 2, but a supplementary table is available from the corresponding author, upon request.

## Discussion

This study sought to investigate the proportion of the Norwegian population that had an alcohol conversation in routine health care during the previous 12 months, to explore individual’s experiences and perceived effects of these conversations, and to investigate the proportion of risky drinkers in the population. The results show that almost one third of the respondents were classified as risky drinkers. The proportion experiencing alcohol conversations in health care was small, and decreasing with increased age. Men more frequently had alcohol conversations than women did, but risky drinkers did not have more alcohol conversations. Risky drinkers more frequently found such conversations awkward or judgmental. Advice on how to cut down on drinking was seldom a part of an alcohol conversation.

The majority of the respondents (79.4%) had visited health care during the previous 12 months, somewhat higher than in Sweden (68.8%) and Netherlands (72.7%) [14]. The proportion classified as risky drinkers (30.5%) is comparable to that found in Sweden and the Netherlands (28.2 and 29.7%) [14]. According to a European report, just over 30% of Norwegian adults reported heavy episodic drinking the past 30 days [24]. A recent national report found risky or harmful alcohol consumption in 13.2% of adults, assessed by full AUDIT score 8 or higher [25]. These figures are not directly comparable, as heavy episodic drinking alone without indication of harm or addiction will result in an AUDIT score below 8.

We found no significant association between education and alcohol consumption, in contrast to other studies demonstrating an association between higher educational attainment and daily alcohol consumption and problem drinking, particularly for women [26]. Previous research indicates an inverse distribution of alcohol consumption and health harms, with higher consumption in groups with higher SES, and a disproportionate burden of negative alcohol-related consequences in groups with low SES [27, 28]. We did not find any significant association between SES and alcohol conversations, which in part may be caused by the fact that Norway is an affluent and relatively equitable country, and in part because we have investigated few factors related to SES [29].

We found that the proportion of respondents with alcohol conversations decreased with age. In contrast, previous research and national data indicates that older adults are more susceptible to negative health consequences of alcohol, and see their primary care physician more frequently than younger adults, thus creating more reasons and more opportunities for addressing alcohol [30, 31]. This is in line with evidence indicating a higher threshold for initiating alcohol conversations with older adults [32].

Our findings highlight the gender difference, both in terms of health care visits and in alcohol consumption. Even though men seek health care less frequently than women do, they experience alcohol conversations more frequently when seeking health care. The likelihood for a woman in our sample to have had an alcohol conversation was almost half (OR 0.56, *p* = 0.004), compared to men. The same difference was found in Sweden and in Netherland, albeit with a smaller gender difference than in Norway [OR for women compared to men was 0.86 (ns) in Sweden, and 0.81 (*p* = 0.015) in Netherlands] [14, 15].

Respondents on parental leave showed an eight-fold increase in the likelihood of having had an alcohol conversation. This is in line with Swedish data and Dutch data [14, 15]. In our sample, respondents on parental leave are few (*n* = 14) and exclusively female, indicating that this may be related to routinely addressing alcohol in the early stages of pregnancy. Primary care physicians and midwives are jointly responsible for antenatal care in Norway [19, 33].

While risky drinkers were not more likely to have experienced an alcohol conversation compared to low risk drinkers or abstainers, their conversations were significantly longer. However, a recent review found no clear evidence that increased exposure (counselling-based vs. advice-based interventions) resulted in larger effect sizes [7]. Our results show that while a majority of alcohol conversations include questions about the patient’s drinking, only around one in ten experiences an exploration of their willingness to change, or receives actual advice on how to cut down on alcohol.

The results suggest that risky drinkers find alcohol conversations awkward (14.3%, *p* = 0.019) and judgmental (19.6%, *p* = 0.045), and as many as 17.9% (*p* = 0.023) of risky drinkers reported increasing their drinking afterwards. This is in contrast to similar studies, where 5.2% (n.s.) of drinkers in Sweden and 2.1% (n.s.) in the Netherlands, reported an increase in their consumption [14]. We do not know whether respondents increased their drinking, or whether this response was triggered by a negative reaction to the alcohol conversation or to this questionnaire.

The same difference between Norway and Sweden and the Netherlands is seen in risky drinkers reports on how the conversations made them feel. In Norway, 14.3% (*p* = 0.019) found it awkward, in contrast to 2.2% (n.s.) in Sweden and 8.3% in the Netherlands (n.s.), and 19.6% (*p* = 0.045) in Norway found it judgmental, compared to 7.1% (n.s.) in Sweden and 3.1% (n.s.) in Netherlands [14]. Interestingly, a study comparing social norms in 33 countries found that Norway had tighter social norms than other European countries, only surpassed by countries in South East Asia [34]. This may partly explain why risky drinkers in Norway perceive alcohol conversations more negatively.

Feelings of shame and guilt may be invoked by health care personnel and their manner of asking about topics which are perceived to be sensitive, but patients with an alcohol problem may bring shameful feelings to the health care visit [35]. Aiming for a non-judgmental conversation is necessary, but perhaps not sufficient. While discussing alcohol consumption with a patient often is straightforward, awareness of the difficult feelings a patient may harbour, can help the patient accept these feelings, and thus support change [35, 36].

Unlike Norway, Sweden has implemented a large-scale national project for brief alcohol interventions in primary health care (The Risk Drinking project) based on motivational interviewing, a strategy for non-judgmental conversations on lifestyle changes [37, 38]. In addition, there are notable differences in alcohol culture with approximately twice as many in Norway (about 80%) compared to Sweden reporting having been drunk the past 12 months, in spite of a slightly higher per capita alcohol consumption in Sweden [39, 40].

### Strengths and Limitations

This study is based on a large, representative panel in all age groups, using a validated screening instrument (AUDIT-C) to assess drinking status, and a questionnaire previously employed in comparable national surveys in Europe, enabling comparisons with international studies. These studies provide new knowledge on how alcohol conversations are perceived, important for improving quality and reach of brief alcohol interventions in health care.

The study also has important limitations. We do not know what happens in the conversations or afterwards, and we do not know who initiated the conversation. All data are self-reported, and the cross-sectional design does not allow causal inferences. While the panels are representative on demographic variables, we do not know how representative they are on drinking habits and experiences with alcohol conversations. Additionally, non-responders might differ from responders. Our sample was relatively more educated than the general population in Norway; approximately 70% of our sample were educated to university-level, which is twice the national rate [41]. This suggests that the sample is skewed towards higher educational level, and probably higher alcohol consumption, although we lack relevant population data to confirm this [26]. The high proportion having visited health care indicates that our sample it is not healthier, which supports the representativeness. However, we lack data on health status of the respondents.

### Implications for Policy, Practice and Future Research

We need a better understanding of how people drink and how they think about drinking, and of how risky drinkers experience alcohol conversations in health care. Alcohol should be addressed as part of the regular clinical work, and exploration of willingness to change and advice on how to cut down, should be an integral part of alcohol conversations for all risky drinkers. Although the majority of risky drinkers did not find the conversation awkward or judgmental, clinicians need to know that a substantial proportion of risky drinkers may perceive the conversations as awkward and judgmental, and strive to find ways to overcome this. Future research and quality improvement should be based upon an improved understanding of patient’s experiences and perspectives, to enable more targeted and effective interventions [42].

### Conclusion

Our study provides important insight in the different aspects of risky drinker’s perceptions of alcohol conversations. The proportion experiencing an alcohol conversation in routine health care was low, also among risky drinkers, and risky drinkers more frequently experienced the conversations as judgmental. Older adults had a significant lower likelihood of experiencing an alcohol conversation. Addressing alcohol should be an integrated task in health care, and especially for groups more at risk for alcohol-related health problems, due to increased consumption and/or increased vulnerability for alcohol. This is particularly relevant for older adults, as physiological changes, health problems and medications increase their vulnerability for alcohol. To increase the normalisation of addressing alcohol in health care, more research is needed on the development and implementation of sensitive and acceptable interventions targeting at-risk patients. Future research should address both implementation factors and acceptability of brief alcohol interventions.

## References

[1] Knudsen AK Kinge JM Skirbekk V Vollset SE. Sykdomsbyrde i Norge 1990-2013 Resultater fra Global Burden of Diseases, Injuries and Risk Factors Study 2013 (GBD 2013). Oslo, Norway: Folkehelseinstituttet (2016).

[2] van Beurden I Anderson P Akkermans RP Grol RPTM Wensing M Laurant MGH. Involvement of General Practitioners in Managing Alcohol Problems: a Randomized Controlled Trial of a Tailored Improvement Programme. Addiction (2012) 107(9):1601–11. 10.1111/j.1360-0443.2012.03868.x 22372573

[3] Kaner E Bland M Cassidy P Coulton S Dale V Deluca P Effectiveness of Screening and Brief Alcohol Intervention in Primary Care (SIPS Trial): Pragmatic Cluster Randomised Controlled Trial. BMJ (2013) 346:e8501. 10.1136/bmj.e8501 23303891 PMC3541471

[4] Drummond C Deluca P Coulton S Bland M Cassidy P Crawford M The Effectiveness of Alcohol Screening and Brief Intervention in Emergency Departments: a Multicentre Pragmatic Cluster Randomized Controlled Trial. PLoS One (2014) 9(6):e99463. 10.1371/journal.pone.0099463 24963731 PMC4070907

[5] Angus C Latimer N Preston L Li J Purshouse R. What Are the Implications for Policy Makers? A Systematic Review of the Cost-Effectiveness of Screening and Brief Interventions for Alcohol Misuse in Primary Care. Front Psychiatry (2014) 5:114. 10.3389/fpsyt.2014.00114 25225487 PMC4150206

[6] Schmidt CS Schulte B Seo H-N Kuhn S O'Donnell A Kriston L Meta-analysis on the Effectiveness of Alcohol Screening with Brief Interventions for Patients in Emergency Care Settings. Addiction (2016) 111(5):783–94. 10.1111/add.13263 26637990

[7] Kaner EF Beyer FR Muirhead C Campbell F Pienaar ED Bertholet N Effectiveness of Brief Alcohol Interventions in Primary Care Populations. Cochrane Database Syst Rev (2018) 2:CD004148. 10.1002/14651858.CD004148.pub4 29476653 PMC6491186

[8] Hellum R Bjerregaard L Nielsen AS. Factors Influencing whether Nurses Talk to Somatic Patients about Their Alcohol Consumption. Nordic Stud Alcohol Drugs (2016) 33(4):415–36. 10.1515/nsad-2016-0034

[9] Derges J Kidger J Fox F Campbell R Kaner E Hickman M. Alcohol Screening and Brief Interventions for Adults and Young People in Health and Community-Based Settings: a Qualitative Systematic Literature Review. BMC Public Health (2017) 17(1):562. 10.1186/s12889-017-4476-4 28599632 PMC5466741

[10] Broyles LM Rodriguez KL Kraemer KL Sevick MA Price PA Gordon AJ. A Qualitative Study of Anticipated Barriers and Facilitators to the Implementation of Nurse-Delivered Alcohol Screening, Brief Intervention, and Referral to Treatment for Hospitalized Patients in a Veterans Affairs Medical center. Addict Sci Clin Pract (2012) 7:7. 10.1186/1940-0640-7-7 23186245 PMC3533719

[11] Beich A Gannik D Malterud K. Screening and Brief Intervention for Excessive Alcohol Use: Qualitative Interview Study of the Experiences of General Practitioners. BMJ (2002) 325(7369):870. 10.1136/bmj.325.7369.870 12386040 PMC129636

[12] Lid TG Nesvåg S Meland E. When General Practitioners Talk about Alcohol: Exploring Facilitating and Hampering Factors for Pragmatic Case Finding. Scand J Public Health (2015) 43(2):153–8. 10.1177/1403494814565129 25564115

[13] O'Donnell A Abidi L Brown J Karlsson N Nilsen P Roback K Beliefs and Attitudes about Addressing Alcohol Consumption in Health Care: a Population Survey in England. BMC Public Health (2018) 18(1):391. 10.1186/s12889-018-5275-2 29562901 PMC5863360

[14] Abidi L Nilsen P Karlsson N Skagerström J O’Donnell A. Conversations about Alcohol in Healthcare - Cross-Sectional Surveys in the Netherlands and Sweden. BMC Public Health (2020) 20(1):283. 10.1186/s12889-020-8367-8 32131793 PMC7057588

[15] Karlsson NE O’Donnell AJ Abidi L Skagerström JME Nilsen PM. Addressing Alcohol in Routine Healthcare in Sweden-population-based Surveys in 2010 and 2017. Eur J Public Health (2019) 29(4):748–53. 10.1093/eurpub/ckz057 31348833

[16] Lid TG Tvedt H Idsøe BN Hustvedt IB Nesvåg S. Is it Acceptable to Be Asked about Alcohol Habits when Admitted to a Somatic Hospital ward? Sykepleien Forskning (2020) 15:e-80932. 10.4220/Sykepleienf.2020.80932

[17] SSB. [Alcohol sold] Alkoholomsetning: Statistics Norway; 2018 (2018). [Alcohol sold in Norway] Statistikk på alkoholomsetning i Norge]. Available from: https://www.ssb.no/varehandel-og-tjenesteyting/statistikker/alkohol (Accessed March 21, 2018).

[18] Vinmonopolet. Annual Report 2017. Oslo, Norway: Vinmonopolet; 2018.

[19] Helsedirektoratet, editor. [Prenatal care] Svangerskapsomsorgen. Oslo, Norway: Helsedirektoratet (2018).

[20] Helsedirektoratet. [Co-occuring substance use disorder and mental disorder] Samtidig ruslidelse og psykisk lidelse (ROP-lidelser). In: Directorate H, editor. Oslo, Norway: Helsedirektoratet (2012).

[21] HOD. [Commission Document to Hospitals] Oppdragsdokument 2013 Helse Vest RHF. in Services DoHaC. Oslo, Norway: Helse- og omsorgsdepartementet (2013).

[22] Karlsson N Skagerström J O’Donnell A Abidi L Thomas K Nilsen P Public Perceptions of How Alcohol Consumption Is Dealt with in Swedish and Norwegian Health Care. Nordic Stud Alcohol Drugs (2021) 38: 1455072520985981. 10.1177/1455072520985981 PMC8899254 35310609

[23] Bush K Kivlahan DR McDonell MB Fihn SD Bradley KAJA. The AUDIT Alcohol Consumption Questions (AUDIT-C)An Effective Brief Screening Test for Problem Drinking. Arch Intern Med (1998) 158(16):1789–95. 10.1001/archinte.158.16.1789 9738608

[24] OECD. Health at a Glance: Europe 2020. Paris: European UnionOECD (2020).

[25] NIPH. [Alcohol use in the adult population] Alkoholbruk i Den Voksne Befolkningen Oslo: Norwegian Institute of Public Health (2020). Available from: https://www.fhi.no/nettpub/alkoholinorge/omsetning-og-bruk/alkoholbruk-i-den-voksne-befolkningen/ (Accessed March 23, 2020).

[26] Grittner U Kuntsche S Gmel G Bloomfield K. Alcohol Consumption and Social Inequality at the Individual and Country Levels-Results from an International Study. Eur J Public Health (2013) 23(2):332–9. 10.1093/eurpub/cks044 22562712 PMC3610336

[27] Collins SE. Associations between Socioeconomic Factors and Alcohol Outcomes. Alcohol Res Curr Rev (2016) 38(1):83. 10.35946/arcr.v38.1.11 PMC4872618 27159815

[28] Beard E Brown J West R Kaner E Meier P Michie S. Associations between Socio-Economic Factors and Alcohol Consumption: A Population Survey of Adults in England. PLoS One (2019) 14(2):e0209442. 10.1371/journal.pone.0209442 30716098 PMC6361426

[29] OECD. Norway Economic Snapshot: OECD; (2021) [Economic snapshot of Norway]. Available from: https://www.oecd.org/economy/norway-economic-snapshot/ (Accessed May 21, 2021).

[30] Norway S. (2020). GPs and emergency primary health care [Web page]. Oslo, Norway, Statistics Norway, Official statistics for Norway]. Available from: https://www.ssb.no/en/helse/statistikker/fastlegetj (Accessed July 14, 2020).

[31] Taylor C Jones KA Dening T. Detecting Alcohol Problems in Older Adults: Can We Do Better? Int Psychogeriatr (2014) 26(11):1755–66. 10.1017/s1041610214001641 25115642

[32] Bareham BK Kaner E Spencer L Hanratty B. Health and Social Care Providers' Perspectives of Older People's Drinking: a Systematic Review and Thematic Synthesis of Qualitative Studies. Age and ageing (2020) 49(3):453–67. 10.1093/ageing/afaa005 PMC7187873 32080741

[33] Hemminki E Blondel B. Antenatal Care in Europe: Varying Ways of Providing High-Coverage Services. Eur J Obstet Gynecol Reprod Biol (2001) 94(1):145–8. 10.1016/s0301-2115(00)00304-3 11134840

[34] Gelfand MJ Raver JL Nishii L Leslie LM Lun J Lim BC Differences between Tight and Loose Cultures: A 33-nation Study. Science (2011) 332(6033):1100–4. 10.1126/science.1197754 21617077

[35] Taylor DR. "You Are Now Entering a Guilt-free Zone". N Engl J Med (2020) 383(22):2103–5. 10.1056/nejmp2003459 33253508

[36] Guassora AD Reventlow S Malterud K. Shame, Honor and Responsibility in Clinical Dialog about Lifestyle Issues: a Qualitative Study about Patients' Presentations of Self. Patient Edu Couns (2014) 97(2):195–9. 10.1016/j.pec.2014.08.003 25154338

[37] Nilsen P Wåhlin S Heather N. Implementing Brief Interventions in Health Care: Lessons Learned from the Swedish Risk Drinking Project. Ijerph (2011) 8(9):3609–27. 10.3390/ijerph8093609 22016706 PMC3194107

[38] Lundahl B Moleni T Burke BL Butters R Tollefson D Butler C Motivational Interviewing in Medical Care Settings: a Systematic Review and Meta-Analysis of Randomized Controlled Trials. Patient Edu Couns (2013) 93(2):157–68. 10.1016/j.pec.2013.07.012 24001658

[39] Moskalewicz J Room R Thom B. Comparative Monitoring of Alcohol Epidemiology across the EU. Baseline Assessment and Suggestions for Future Action Synthesis Report Warsaw. Warsaw, Poland: PARPA-The State Agency for Prevention of Alcohol Related Problems (2016).

[40] World Health Organization. Non-communicable Diseases Country Profiles 2011. France: World Health Organization (2011).

[41] Norway S. Educational Attainment of the Population Oslo: Statistics Norway (2020). Available at: https://www.ssb.no/en/utdanning/statistikker/utniv (Accessed July 15, 2020).

[42] O'Donnell A Hanratty B Schulte B Kaner E. Patients' Experiences of Alcohol Screening and Advice in Primary Care: a Qualitative Study. BMC Fam Pract (2020) 21(1):68. 10.1186/s12875-020-01142-9 32321440 PMC7178930

